# Supplementary material for: Metabolite trafficking enables membrane-impermeable-terpene secretion by yeast
Source: Nat Commun. 2022 May 11;13:2605. doi: 10.1038/s41467-022-30312-9 (PMC9095633; doi:10.1038/s41467-022-30312-9)
Supplement: Supplementary file 1 — Supplementary Information [file 41467_2022_30312_MOESM1_ESM.pdf]

Supplementary Information:

Metabolite trafficking enables membrane-impermeable-terpene secretion by yeast

So-Hee Son[1,2,†], Jae-Eung Kim[1,†], Gyuri Park[3], Young-Joon Ko[1], Bong Hyun Sung[4], Jongcheol Seo[5], Seung Soo Oh[2,3,6,*], and Ju Young Lee[1,*]

[1] Research Center for Bio-based Chemistry, Korea Research Institute of Chemical Technology (KRICT), 406-30, Jongga-ro, Jung-gu, Ulsan 44429, Republic of Korea

[2] School of Interdisciplinary Bioscience and Bioengineering, Pohang University of Science and Technology (POSTECH), Pohang, Gyeongbuk 37673, Republic of Korea

[3] Department of Materials Science and Engineering, Pohang University of Science and Technology (POSTECH), Pohang, Gyeongbuk 37673, Republic of Korea

[4] Synthetic Biology and Bioengineering Research Center, Korea Research Institute of Bioscience and Biotechnology (KRIBB), Daejeon 34141, Republic of Korea

[5] Department of Chemistry, Pohang University of Science and Technology (POSTECH), Pohang, Gyeongbuk 37673, Republic of Korea

[6] Institute for Convergence Research and Education in Advanced Technology (I-CREATE), Yonsei University, Incheon 21983, Republic of Korea

[†] These authors contributed equally to this work.

[*] **Corresponding authors:**

Dr. Ju Young Lee

Phone: +82-52-241-6325

Fax: +82-52-241-6359

E-mail: juylee@krict.re.kr

Prof. Seung Soo Oh

Phone: +82-54-279-2144

Fax: +82-54-279-2399

E-mail: seungsoo@postech.ac.kr

Table of Contents

Supplementary Fig. 1: Crystal structure and amino acid sequence of the supernatant protein factor (SPF)

Supplementary Fig. 2: Schematic representation of the post-translational and co-translational translocation that we exploited for metabolite secretion

Supplementary Fig. 3: HPLC chromatograms of secreted squalene (A) and β-carotene (B)

Supplementary Fig. 4: Quantitative measurements of secreted and accumulated squalene.

Supplementary Fig. 5: Subcellular localization of tSPF-GFP fused with different signal peptides

Supplementary Fig. 6: Western blot analysis of cell lysate (top) and culture supernatant (bottom) by overexpression of Suc2-GFP

Supplementary Fig. 7: Semi-continuous fermentation system using SQ strain

Supplementary Fig. 8: The tSPF-mediated β-carotene secretion

Supplementary Fig. 9: Predicted complex structures of SPF A chain and terpene ligands

Supplementary Fig. 10: The predicted peak positions of tSPF ions with various charge states (A) and the experimental ESI-mass spectrum of solution containing tSPF and squalene (B)

Supplementary Fig. 11: *In vivo* and *in vitro* evaluation of cluster formation by multiple tSPF-squalene complexes using confocal fluorescence microscopy

Supplementary Table 1: Squalene production of tSPF derivative-expressing SQ strain

Supplementary Table 2: 2.3-oxidosqualene production of tSPF derivative-expressing SQ strain

Supplementary Table 3: List of signal peptides in this study

Supplementary Table 4: Semi-continuous fermentation using the SQ strain that overexpresses Suc2-tSPF

Supplementary Table 5: Terpene production of BC strain that overexpresses the tSPF or Suc2-tSPF

Supplementary Table 6: Predicted binding energy for 43 different terpenes to a SPF protein

Supplementary Table 7: Synthetic biology tools and strategies to maximize terpene production and secretion

Supplementary Table 8: List of plasmids and strains in this study

Supplementary Table 9: List of primers in this study

Supplementary Table 10: Comparison of our metabolite trafficking system with membrane transporter approach for terpene secretion

Supplementary Note 1 to 6

Supplementary References

A

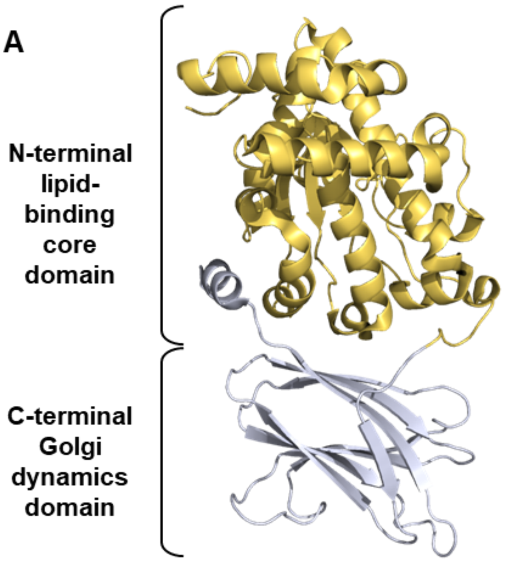

B

SPF sequence (PDB 1O6U)	
MSGRVGDLSPRQKEALAKFRENVQDVLPALPNP	33
DDYFLLRWLRARSFDLQKSEAMLRKHVEFRKQK	66
DIDNIISWQPPEVIQQYLSGGMCGYDLDGCPVW	99
YDIIGPLDAKGLLFSASKQDLLRTKMRECELLL	132
QECAHQTTKLGRKVETITIIYDCEGLGLKHLWK	165
PAVEAYGEFLCMFEENYPETLKRLFVVKAPKLF	198
PVAYNLIKPFLSEDTRKKIMVLGANWKEVLLKH	231
ISPDQVPVEYGGTMTDPDGNPKCKSKINYGGDI	264
PRKYYVRDQVKQQYEHSVQISRGSSHQVEYEIL	297
FPGCVLRWQFMSDGADVGFGIFLKTKMGERQRA	330
GEMTEVLPNQRYNSHLVPEDGTLTCSDPGIYVL	363
RFDNTYSFIHAKKVNFTVEVLLPDKASEEKMKQ	396
LGAGTPK	403

Supplementary fig. 1 Crystal structure and amino acid sequence of supernatant protein factor (SPF). A. Overall structure of SPF as a ribbon diagram. The structure of SPF consists of a lipid-binding core domain (yellow) and a C-terminal Golgi dynamics (GOLD) domain (grey). The figure was prepared using PyMOL (http://www.pymol.org). **B.** Amino acid sequence of SPF (PBD code 1O6U). The amino acid residues of the lipid-binding core domain and the GOLD domain are color-coded in yellow and grey, respectively, in accordance with the colors of the protein structure.

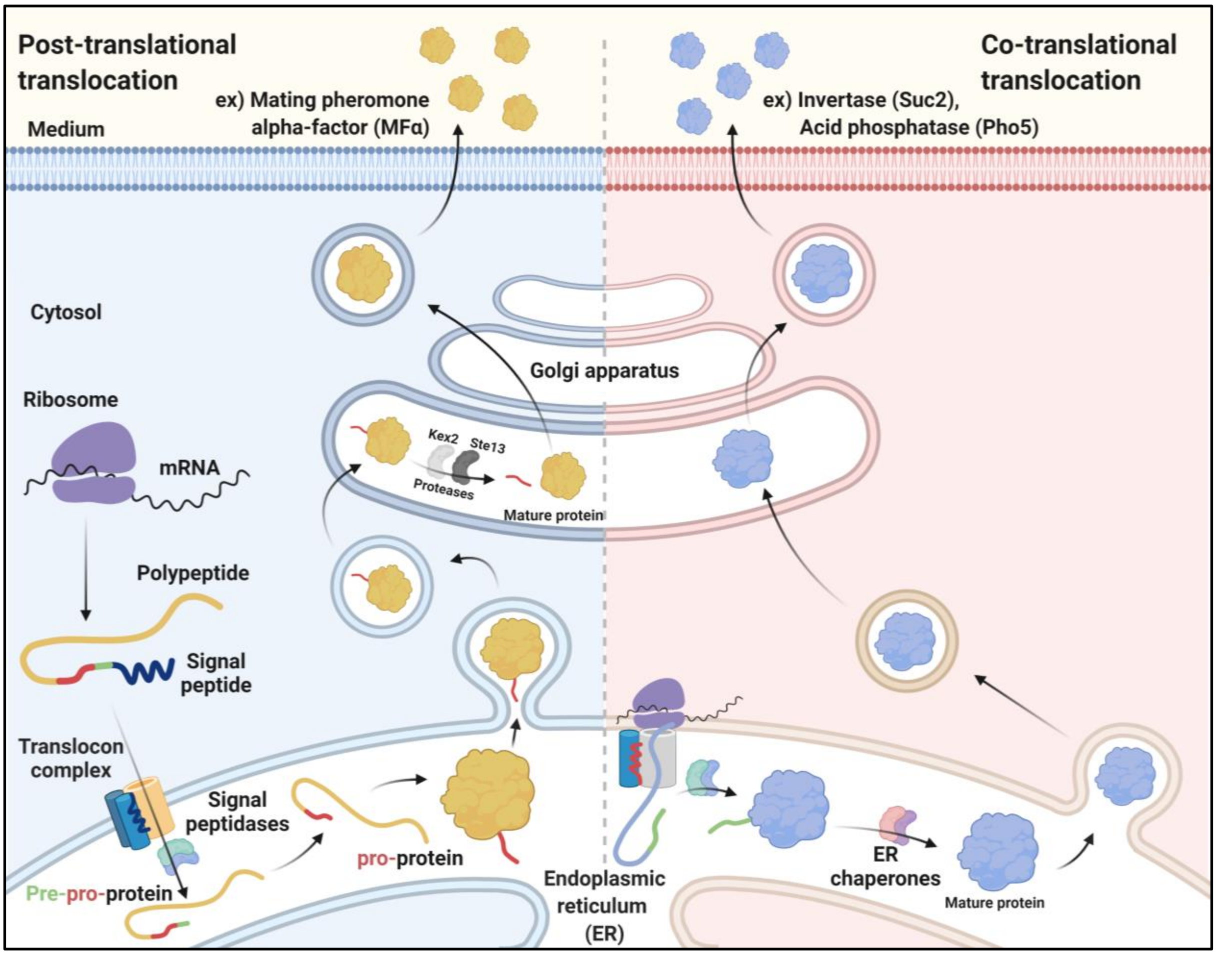

Supplementary fig. 2 Schematic representation of the post-translational and co-translational translocation that we exploited for metabolite secretion. In the post-translational translocation, a complex of ribosome with mRNA initiates and completes the translation of the mRNA in cytosol. Cytosolic chaperones maintain the nascent polypeptide in an unfolded state before it is delivered to a translocon complex in an ER membrane. After cleavage of the signal sequence by signal peptidases, the pre-pro-protein (colored in green, red and yellow, respectively) is released into the ER lumen where it is subject to further processing to form a pro-protein. The “pro” domain is removed in the Golgi apparatus by proteases, such as Kex2 and Ste13, resulting in formation of mature protein, followed by secretion. The yeast α-mating factor (MFα), for example, is secreted into an extracellular milieu by the post-translational translocation. Majority of secretory proteins adopt co-translational translocation. In

contrast to the post-translational translocation that results in the delivery of fully translated polypeptide chains to the ER, the co-translational translocation allows translation of secretory proteins, simultaneously translocating the proteins through a channel in the ER membrane. When the nascent polypeptide is released, it is further modified by ER chaperones to form a mature protein. The mature protein is encapsulated into a transport vesicle, which is next transported to the Golgi apparatus. After a sorting process of secretory proteins in the Golgi, the mature protein is exported into an extracellular medium. Secretory proteins, such as invertase (Suc2) and acid phosphatase (Pho5), are secreted using the co-translational translocation.

A

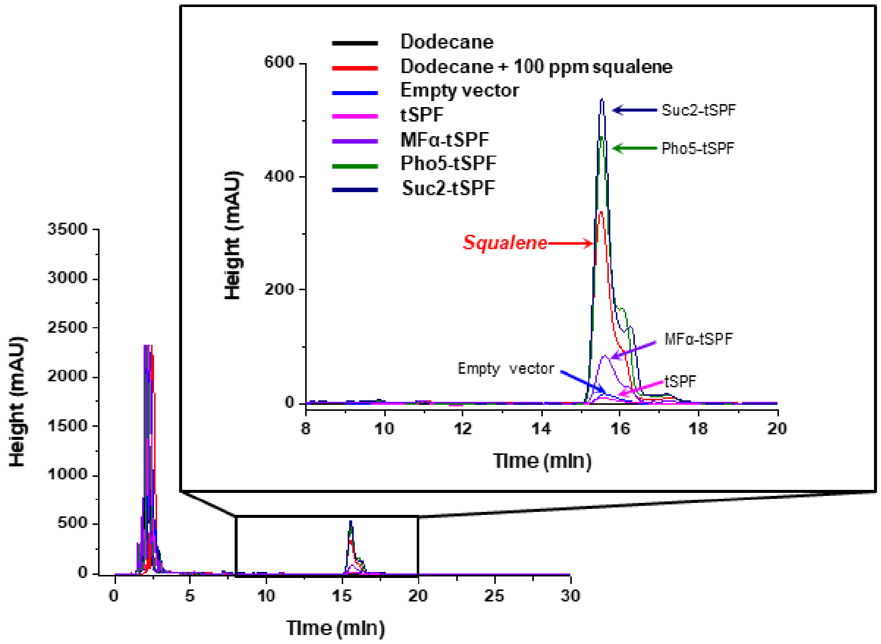

B

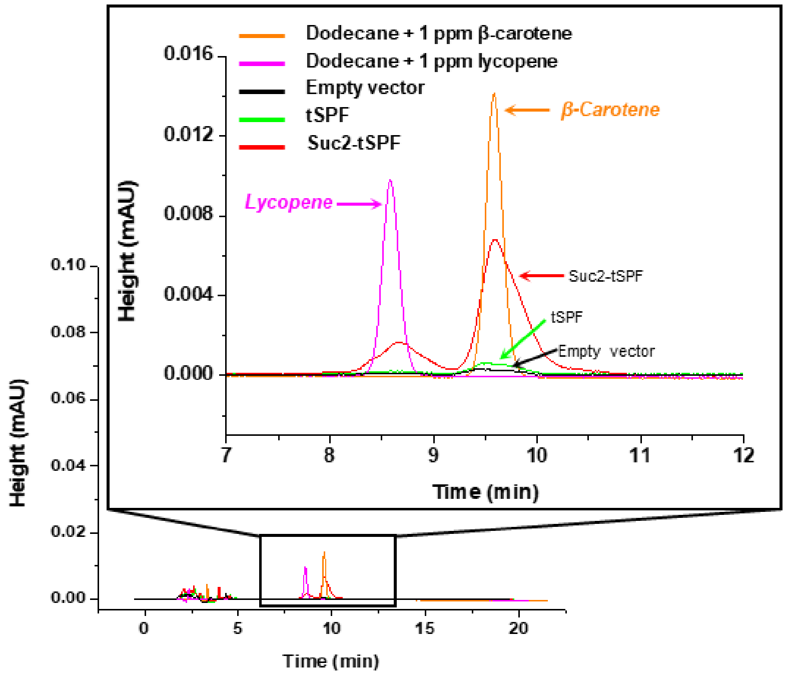

Supplementary fig. 3 HPLC chromatograms of secreted squalene (A) and β-carotene (B). Yeast cells were grown in a defined minimal medium supplemented with 2% (w/v) glucose and 10% (v/v) dodecane at 30°C for 144 h. The retention time was 15.5 min for squalene and 9.5 min for β-carotene. mAU: milli absorbance units.

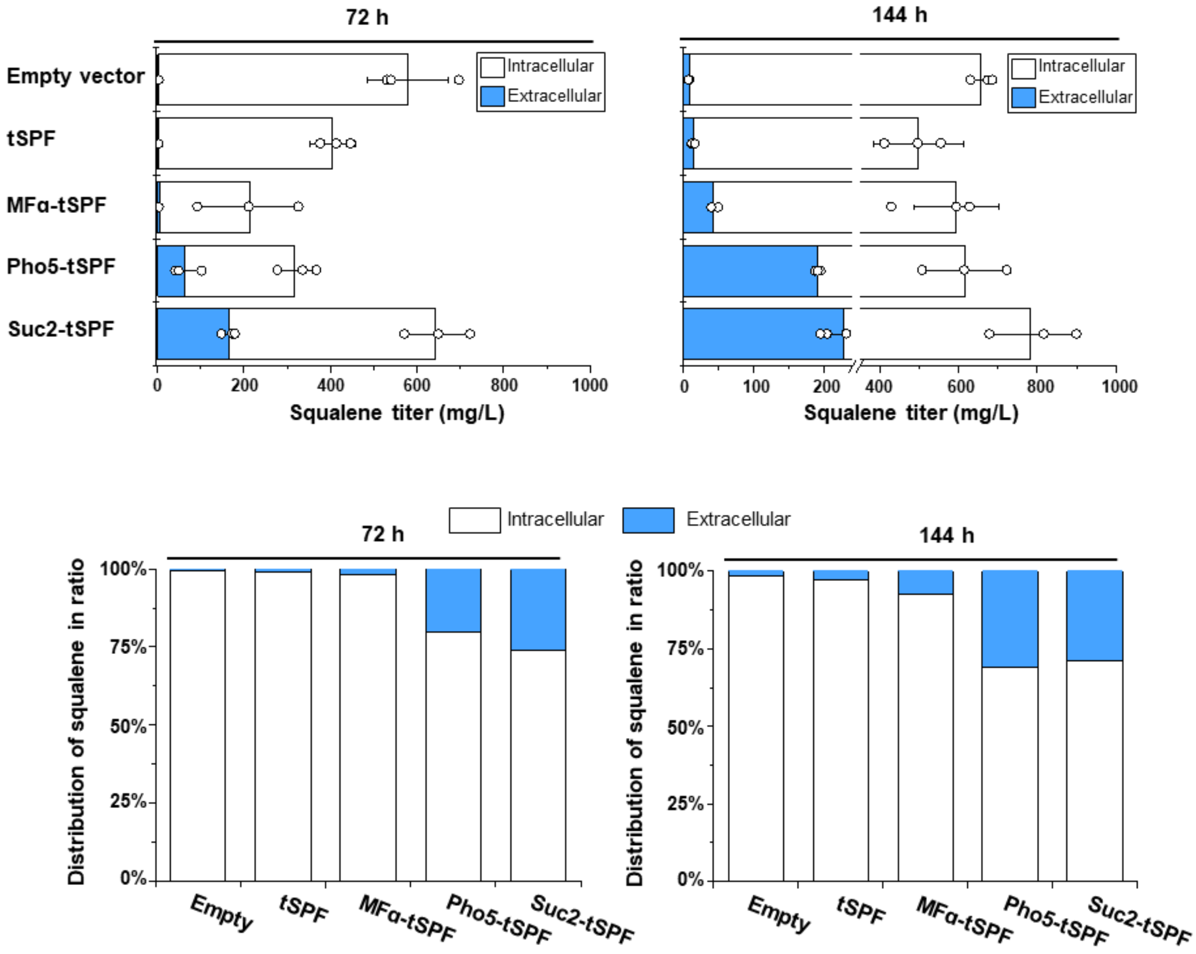

Supplementary fig. 4 Quantitative measurements of secreted and accumulated squalene. Yeast cells were grown in a defined minimal medium supplemented with 2% (w/v) glucose and 10% (v/v) dodecane at 30°C for 144 h. At 72 and 144 h of shake flask cultivation (left and right), extracellular and intracellular squalene was quantified by collection of dodecane phase and disruption of harvested cells, respectively. All data represent the mean of biological triplicates, and error bars indicate the standard deviation.

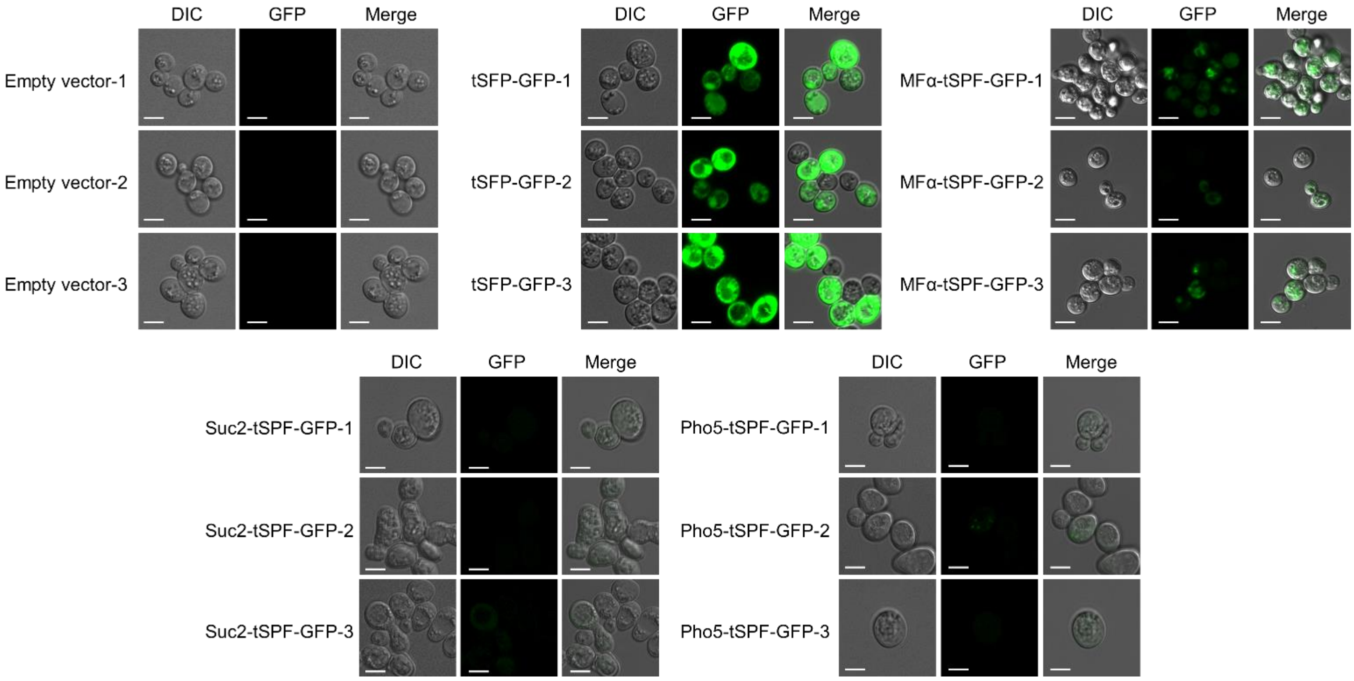

Supplementary fig. 5 Subcellular localization of tSPF-GFP tagged with different signal peptides. The GFP signals were significantly reduced in the cells expressing tSPF-GFP tagged with Suc2 or Pho5 signal peptide, indicating that Suc2-tSPF and Pho5-tSPF were successfully secreted out of cells. Confocal images were processed and analyzed using ZEN imaging software (Zeiss). Scale bar: 5 μm.

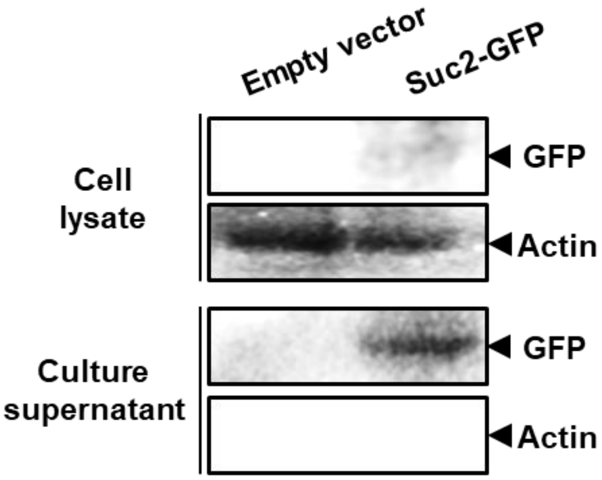

Supplementary fig. 6 Western blot analysis of cell lysate (top) and culture supernatant (bottom) by overexpression of Suc2-GFP. Suc2-GFP was detected using an anti-His antibody. Intracellular endogenous actin from culture supernatant serves as a marker for cell lysis. Source data are provided in the Source Data File.

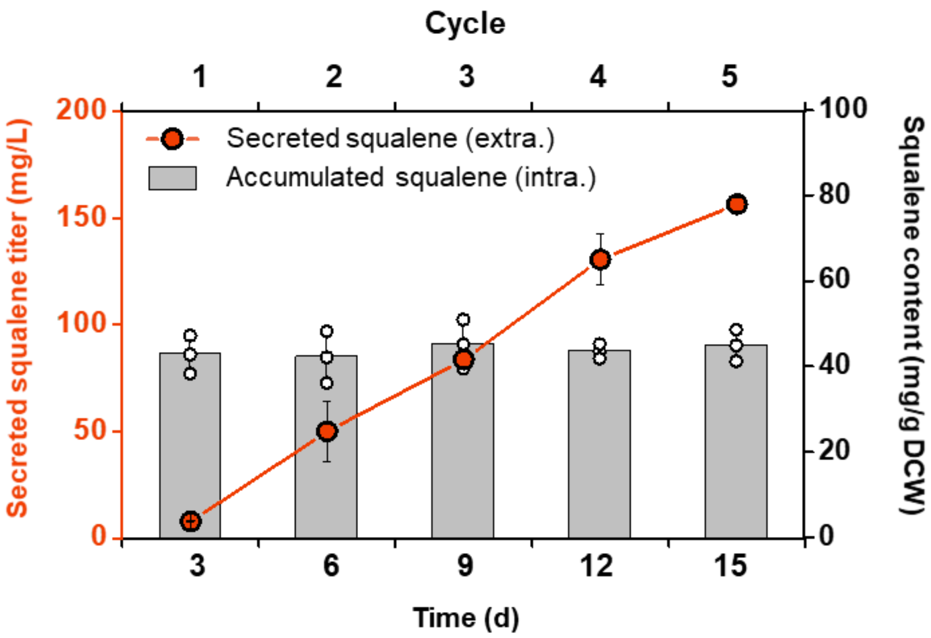

Supplementary fig. 7 Semi-continuous fermentation system using SQ strain. A growth medium was exchanged every 3 days (see method for details) and the levels of intra- and extracellular squalene were monitored over 15 days. All data represent the mean of biological triplicates and error bars show s.d.

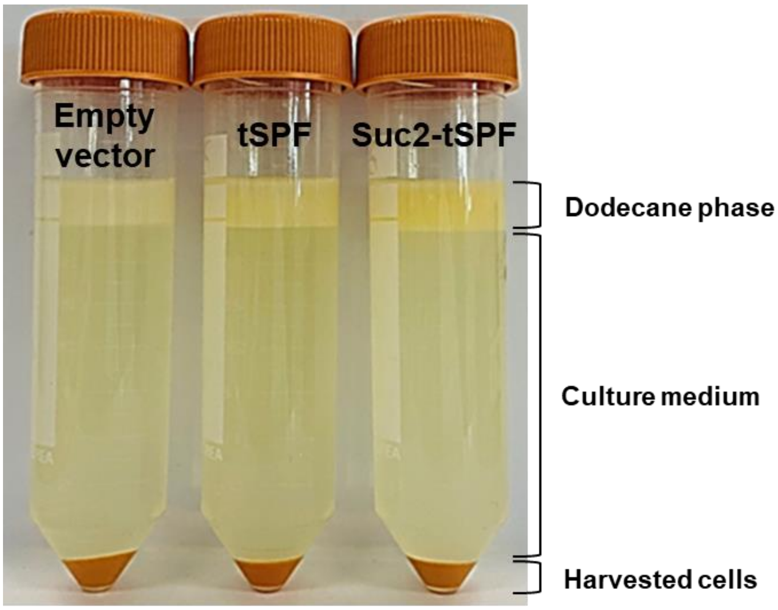

Supplementary fig. 8 The tSPF-mediated β-carotene secretion. The β-carotene-producing strains (BC), concurrent with overexpression of either tSPF or Suc2-tSPF, were grown in a defined minimal medium containing 2% (w/v) glucose and 10% (v/v) dodecane at 30 °C for 144 h. After 144 h cultivation, a noticeable increase of secreted β-carotene in the dodecane layer was observed by using the Suc2-tSPF expressing BC strain. In the culture medium, no β-carotene was detected.

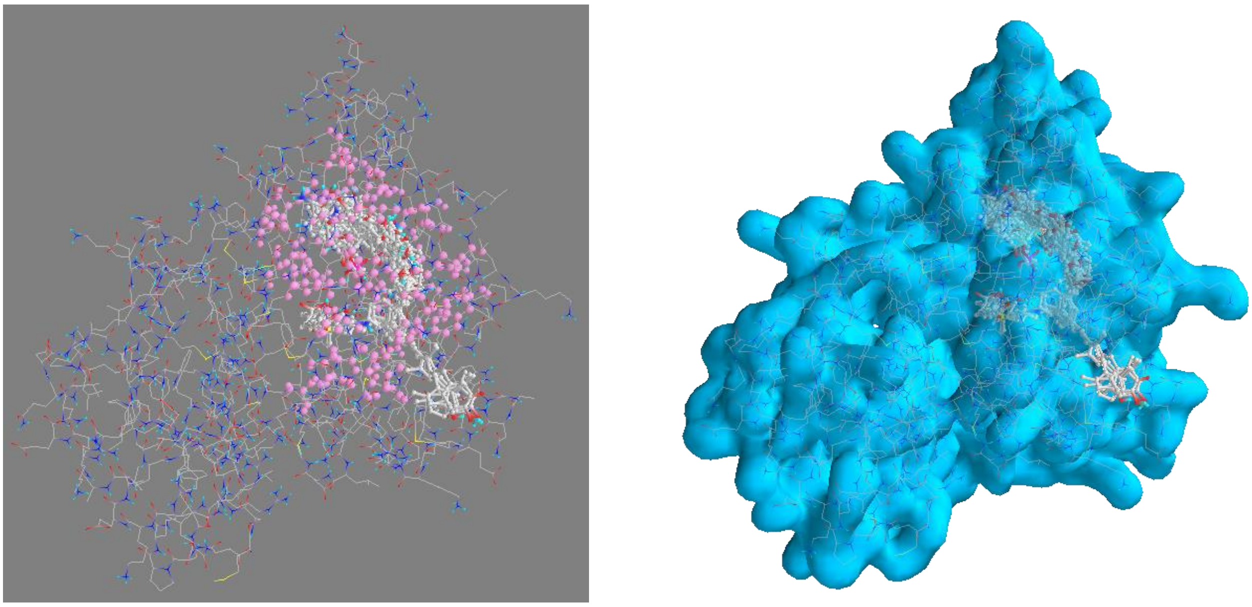

Supplementary fig. 9 Predicted complex structures of SPF A chain and terpene ligands. In a wireframe diagram (left), 43 different terpene ligands are overlappingly positioned within the binding pocket of SPF (PDB ID: 4OMK). The amino acid residues of the binding pocket are shown in magenta. In a surface diagram (right), end groups of several tetra-terpenes such as the isophorone group of zeaxanthin protruded out of the protein surface.

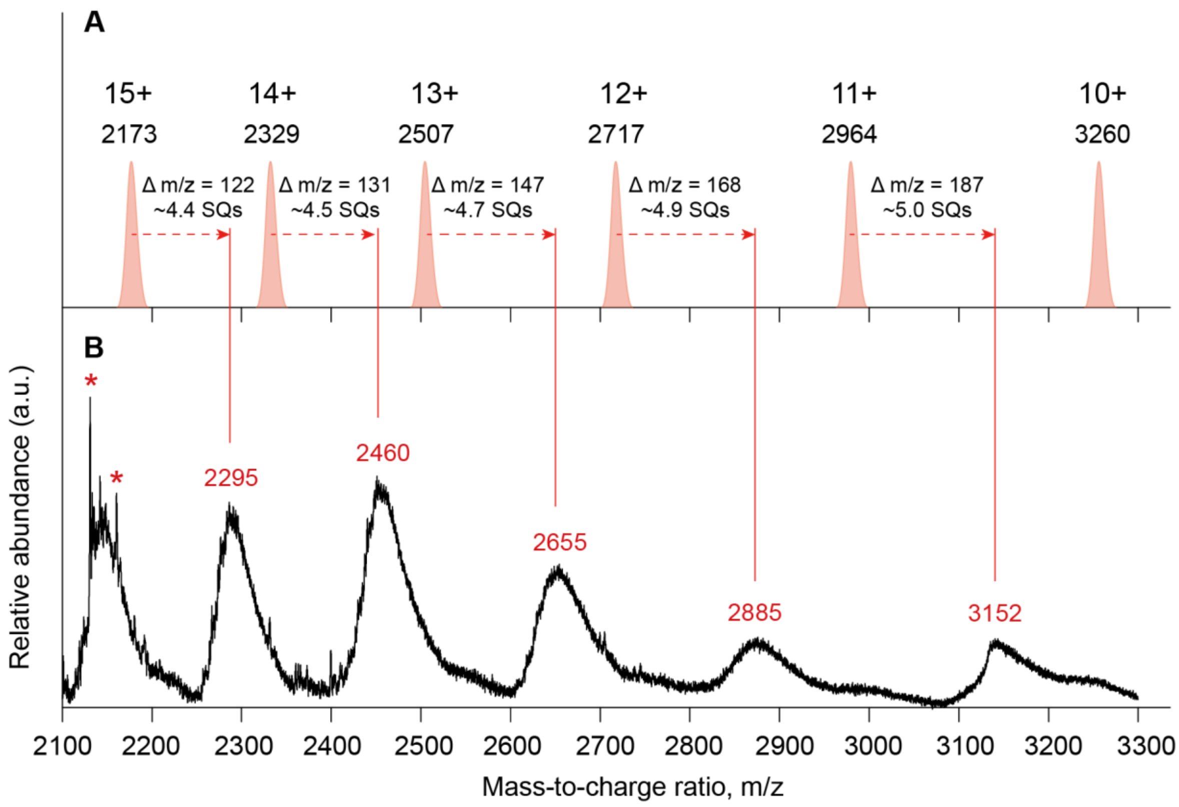

Supplementary fig. 10 The predicted peak positions of tSPF ions with various charge states (A) and the experimental ESI-mass spectrum of solution containing tSPF and squalene (B). The observed mass peaks show the systematic deviations from the tSPF ions, indicating the presence of tSPF-squalene complex with a broad distribution in the binding stoichiometry. The m/z differences determine the average number of bound squalenes, which are 4–5 squalenes per one tSPF on average.

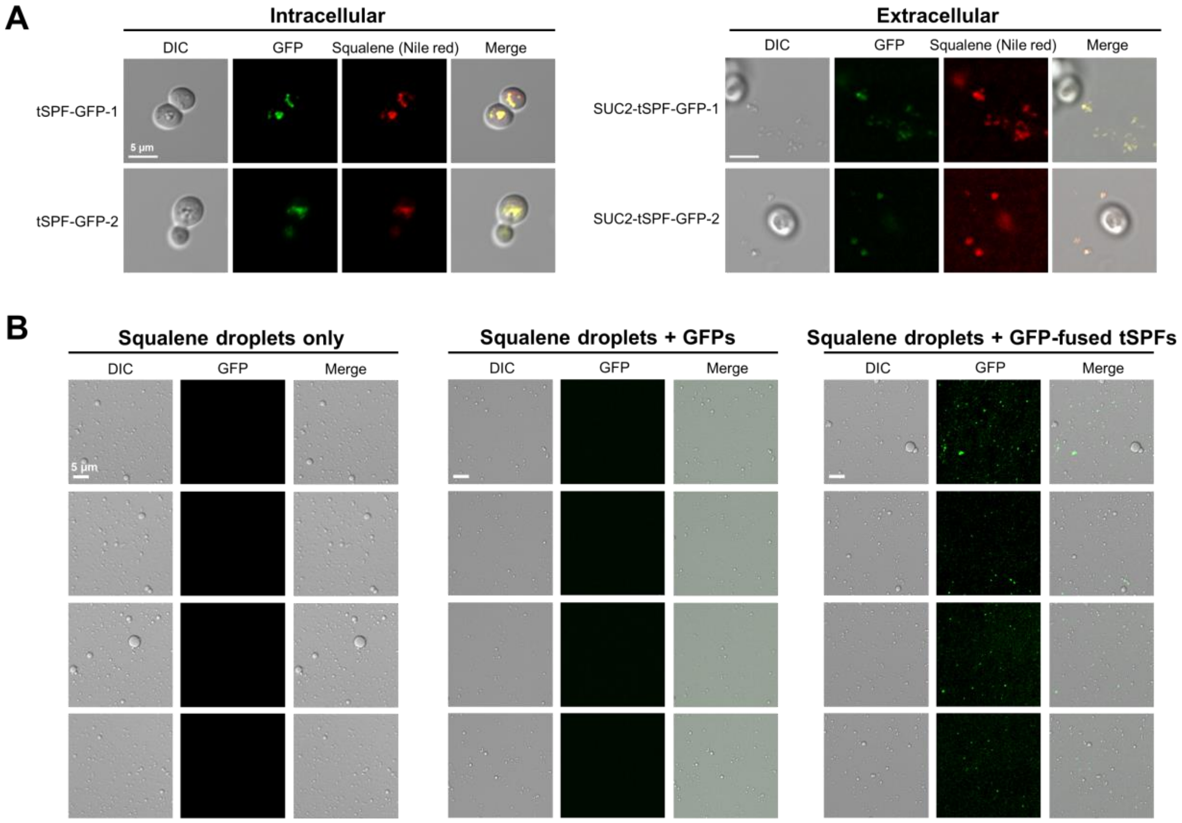

Supplementary fig. 11 *In vivo* and *in vitro* evaluation of cluster formation by multiple tSPF-squalene complexes using confocal fluorescence microscopy. (A) When GFP-fused Suc2-tSPFs were overexpressed by squalene-producing SQ strains, the green fluorescence by the GFP-fused tSPFs and the red one by Nile red-stained squalene were colocalized inside and outside the cells (left and right, respectively), presumably by formation of tSPF-squalene droplet clusters. **(B)** Squalene droplets (100 ~ 400 nm in diameter) were prepared (left) and subsequently mixed with GFP proteins (middle) and GFP-fused tSPFs (right) of which concentrations (~0.1 mg/mL) were 100 times less than that of squalene (~10 mg/mL). Unlike the GFPs only, the GFP-fused tSPFs bound to the squalene droplets as evidenced by their identical locations; from this observation, it is supposed that the tSPF could bind not only to the single squalene molecule but also to its forms of clusters or droplets. For the confocal

fluorescence microscopy observation, squalene was stained with Nile red (excitation at 543 nm and emission at 585 nm), but GFP was fluorescent (excitation at 488 nm and emission at 507 nm). Confocal fluorescence microscopy images were processed and analyzed using ZEN imaging software (Zeiss). Scale bar: 5 μm.

Supplementary Table 1. Squalene production of tSPF derivative-expressing SQ strain.

	Squalene production							
	72 h				144 h			
	**Cell growth (OD_{600})**	**Intracellular squalene (mg/L)**	**Intracellular squalene (mg/g)**	**Extracellular squalene (mg/L)**	**Cell growth (OD_{600})**	**Intracellular squalene (mg/L)**	**Intracellular squalene (mg/g)**	**Extracellular squalene (mg/L)**
Empty vector	20.23 (±2.24)	574.98 (±92.14)	69.32 (±9.60)	3.99 (±1.04)	23.92 (±3.91)	648.03 (±26.69)	66.07 (6.80)	8.73 (±0.76)
tSPF	17.79 (±2.22)	401.51 (±53.63)	55.05 (±5.66)	3.67 (±0.34)	19.91 (±2.68)	485.04 (±109.94)	59.43 (±6.74)	13.82 (±2.61)
Suc2-tSPF	12.98 (±0.46)	476.9 (±75.73)	89.61 (±11.04)	166.62 (±16.44)	13.87 (±1.63)	557.03 (±116.14)	97.93 (±10.37)	226.51 (±20.50)
Pho5-tSPF	11.9 (±1.76)	252.17 (±49.47)	51.68 (±6.98)	64.55 (±33.05)	14.38 (±0.48)	426.85 (±115.13)	72.39 (±16.12)	190.09 (±3.74)
MFα-tSPF	11.44 (±3.28)	210.52 (±117.40)	44.88 (±12.18)	4.21 (±0.26)	17.16 (±1.10)	551.64 (±102.87)	78.42 (±8.45)	43.25 (±5.46)
Suc2-GFP	20.04 (±1.07)	483.8 (±11.74)	48.28 (±0.76)	3.29 (±1.04)	18.94 (±0.03)	563.06 (±2.03)	59.52 (±11.74)	4.71 (±0.62)

Supplementary Table 2. 2.3-oxidosqualene production of tSPF derivative-expressing SQ strain.

	2.3-oxidosqualene production			
	72 h		144 h	
	**Intracellular 2.3-oxidosqualene (mg/L)**	**Intracellular 2.3-oxidosqualene (mg/g)**	**Intracellular 2.3-oxidosqualene (mg/L)**	**Intracellular 2.3-oxidosqualene (mg/g)**
Empty vector	0.62(±0.62)	0.07(±0.07)	0.44(±0.69)	0.06(±0.07)
tSPF	0.24(±0.42)	0.03(±0.06)	ND	ND
Suc2-tSPF	0.88(±0.27)	0.15(±0.04)	0.74(±0.00)	0.05(±0.06)
Pho5-tSPF	0.35(±0.35)	0.06(±0.06)	0.22(±0.34)	0.11(±0.00)
MFα-tSPF	0.42(±0.52)	0.07(±0.09)	ND	ND
Suc2-GFP	ND	ND	ND	ND

*ND, not detected.

Supplementary Table 3. List of signal peptides in this study.

Signal peptide	Sequence	Length of sequence (amino acids)
Suc2	MLLQAFLFLLAGFAAKISA	19
Pho5	MFKSVVYSILAASLANA	17
MFα	MRFPSIFTAVLFAASSALAAPVNTTTEDETAQIPAEAVIGYSDLEGDFDVAVLPFSNSTNNGLLFINTTIASIAAKE EGVSLEKREAEA	89

Supplementary Table 4. Semi-continuous fermentation using the SQ strain that overexpresses Suc2-tSPF.

	Squalene production					
	Time (d)	Cell growth (OD_{600})	Intracellular squalene (mg/L)	Intracellular squalene (mg/g)	Extracellular squalene (mg/L)
Empty vector	3	26.86 (±0.93)	576.10 (±31.34)	43.01 (±4.44)	7.89 (±0.26)
	6	42.52 (±1.64)	897.97 (±79.48)	42.40 (±6.05)	50.01 (±14.29)
	9	54.4 (±1.98)	1232.04 (±92.62)	45.44 (±5.74)	83.74 (±3.45)
	12	68.4 (±0.79)	1496.80 (±33.09)	43.78 (±1.68)	130.54 (±11.96)
	15	74.76 (±0.28)	1687.02 (±129.45)	45.14 (±3.70)	156.41 (±3.21)
Suc2-tSPF	3	16.04 (±1.53)	494.39 (±4.65)	62.17 (±7.81)	173.32 (±14.85)
	6	27.84 (±1.47)	828.31 (±48.44)	59.54 (±0.97)	363.85 (±10.65)
	9	41 (±0.42)	1131.53 (±104.86)	55.17 (±4.32)	465.31 (±47.33)
	12	49.28 (±1.47)	1349.04 (±37.75)	54.83 (±3.85)	579.39 (±4.13)
	15	52.6 (±2.66)	1556.72 (±167.24)	59.57 (±10.62)	670.10 (±8.14)

Supplementary Table 5. Terpene production of BC strain that overexpresses tSPF or Suc2-tSPF.

144 h			Empty vector	tSPF	Suc2-tSPF
**Cell growth (OD_{600})**			22.58 (±0.93)	20.36 (±0.06)	18.66 (±0.42)
**Squalene**	Intracellular	(mg/L)	ND	ND	ND
		(mg/g)	ND	ND	ND
	Extracellular	(mg/L)	ND	ND	ND
**Lycopene**	Intracellular	(mg/L)	2.02 (±0.26)	0.56 (±0.01)	0.8 (±0.99)
		(mg/g)	0.25 (±0.02)	0.09(±0.00)	0.10 (±0.12)
	Extracellular	(mg/L)	0.01 (±0.01)	0.02 (±0.00)	0.33 (±0.07)
**β-Carotene**	Intracellular	(mg/L)	9.52 (±0.68)	7.04 (±0.16)	7.65 (±0.01)
		(mg/g)	0.84 (±0.01)	0.69 (±0.00)	0.82 (±0.13)
	Extracellular	(mg/L)	0.06 (±0.05)	0.23 (±0.06)	1.40 (±0.05)

*ND, not detected.

Supplementary Table 6. Predicted binding energy of 43 different terpenes to a SPF protein. The binding energy was calculated using AutoDock Vina in PyRx.

Name	Molecular Formula	Structure	Binding energy
**Pyruvate**	$C_3H_3O_3$	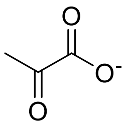	-3.6
**Glutamic acid**	$C_5H_9NO_4$	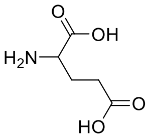	-4.4
**Geraniol**	$C_{10}H_{18}O$	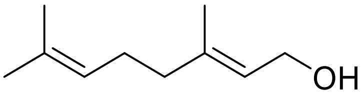	-6.3
**Linalool**	$C_{10}H_{18}O$	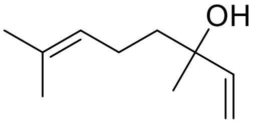	-6.1
**Myrcene**	$C_{10}H_{16}$	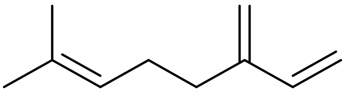	-6.3
**Nerol**	$C_{10}H_{18}O$	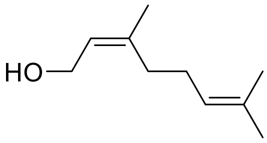	-6.3
**β-Ocimene**	$C_{10}H_{16}$	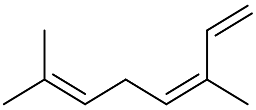	-6.5
**Camphor**	$C_{10}H_{16}O$	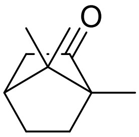	-7.1
**Carvacrol**	$C_{10}H_{14}O$	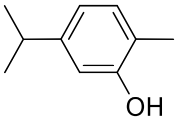	-7.4
**Carvone**	$C_{10}H_{14}O$	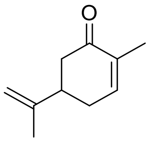	-6.9

**Limonene**	$C_{10}H_{16}$	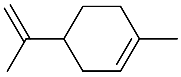	-6.5
**Menthol**	$C_{10}H_{20}O$	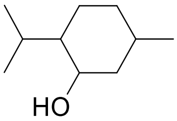	-6.5
**α-Pinene**	$C_{10}H_{16}$	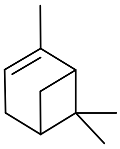	-6.6
**β-Farnesene**	$C_{15}H_{24}$	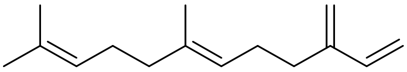	-7.6
**Nerolidol**	$C_{15}H_{26}O$	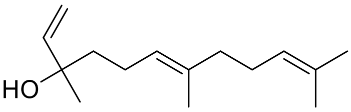	-7.7
**Amorphadiene**	$C_{15}H_{24}$	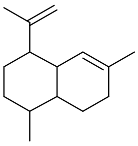	-8.1
**Artemisinin**	$C_{15}H_{22}O_5$	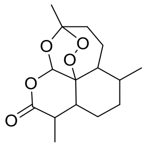	-9.4
**β-Bisabolene**	$C_{15}H_{24}$	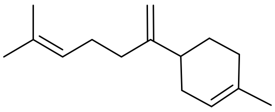	-8.7
**Capsidiol**	$C_{15}H_{24}O_2$	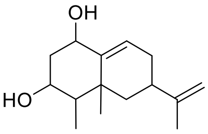	-7.8
**α-Cedrane**	$C_{15}H_{26}$	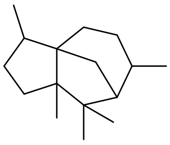	-8.9
**Valencene**	$C_{15}H_{24}$	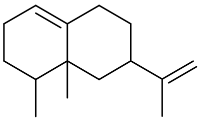	-8.7

**Phytane**	$C_{20}H_{42}$	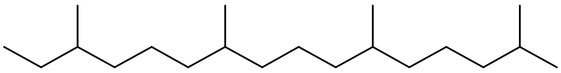	-7.4
**Phytol**	$C_{20}H_{40}O$	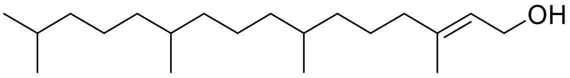	-7.4
**Abietane**	$C_{20}H_{36}$	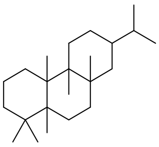	-10.7
**Camphorene**	$C_{20}H_{32}$	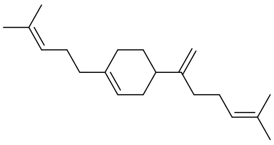	-9.5
**Clerodane**	$C_{20}H_{38}$	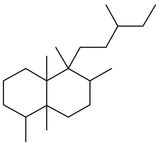	-9.4
**Kaurane**	$C_{20}H_{34}$	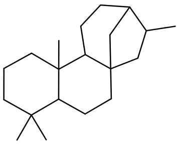	-10.5
**Labdane**	$C_{20}H_{38}$	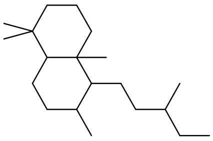	-9.7
**Pimarane**	$C_{20}H_{36}$	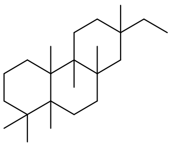	-10.3
**Retinol**	$C_{20}H_{30}O$	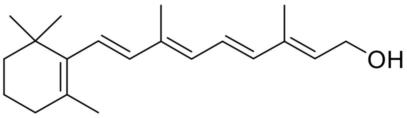	-9.4
**Taxane**	$C_{20}H_{36}$	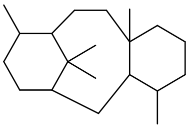	-7.7
**Tigliane**	$C_{20}H_{34}$	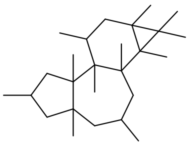	-9.4

**Oxidosqualene**	$C_{30}H_{49}O$	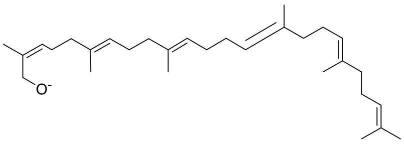	-10.6
**Squalene**	$C_{30}H_{50}$	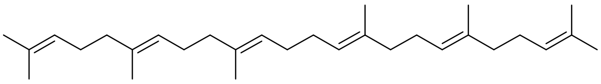	-10.5
**α-Amyrin**	$C_{30}H_{50}O$	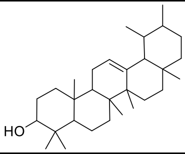	-6.8
**β-Amyrin**	$C_{30}H_{50}O$	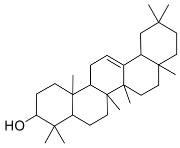	-6.6
**Hopane**	$C_{30}H_{52}$	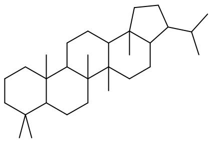	-6.6
**Lanosterol**	$C_{30}H_{50}O$	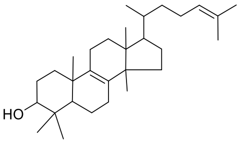	-10.7
**Oleanolic acid**	$C_{30}H_{48}O_3$	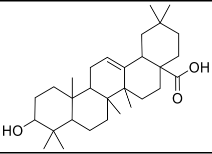	-6.3
**Lycopene**	$C_{40}H_{56}$	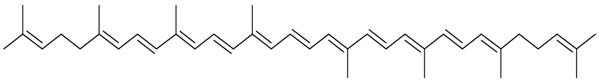	-9.8
**Astaxanthin**	$C_{40}H_{52}O_4$	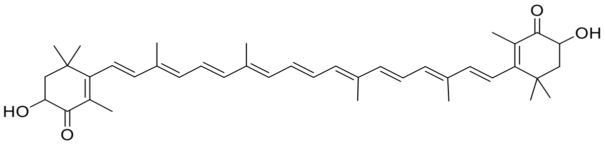	-6.2
**β-Carotene**	$C_{40}H_{56}$	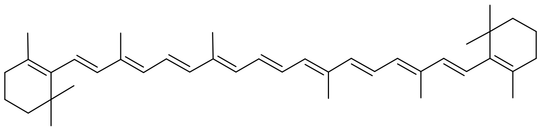	-7.7
**Lutein**	$C_{40}H_{56}O_2$	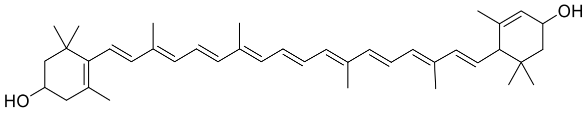	-7.5

**Rubixanthin**	$C_{40}H_{56}O$	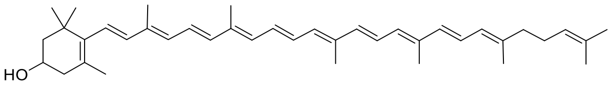	-7.4
**Zeaxanthin**	$C_{40}H_{56}O_2$	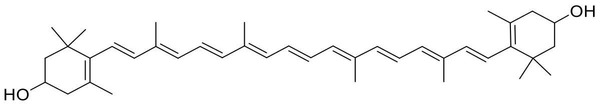	-6.3

Supplementary Table 7. Synthetic biology tools and strategies to maximize terpene production and secretion

Tools	Characteristics
Protein engineering	Structure-based protein engineering can be employed to enhance the specificity and selectivity of proteins for target products. Computational docking simulation can be used to identify the key amino acids that bind to target products in the binding pocket of protein, thereby generating a more favorable protein for the target products.
Plasmid copy number	Gene dosage, as manipulated through plasmid copy number, affects protein expression.
Promoter	Strong/weak, inducible/constitutive, or synthetic hybrid promoters are available, and their regulation is a major influence on protein expression.
Regulatory genetic circuit design	Cells can be programmed using genetic circuits to make product concentrations monitored for metabolic reactions. Protein expression can be tightly coordinated with cell growth or product concentrations.
mRNA stability and structure	The stability and secondary structure of mRNA influences ribosome binding and translational efficiency, thereby affecting protein expression.
Co-overexpression with chaperones	Increased or decreased amounts of several molecular chaperones, foldases, and proteases influence protein yield and quality.
Medium composition	Optimization of growth medium and carbon source can lead to increase of product yield, but decrease of by-product formation.
Process conditions	Temperature, oxygenation, pH and medium osmolarity impact on the production process.
Cellular metabolism rewiring	Rewiring of cellular metabolism by metabolic engineering can maximize metabolic flux towards the desired products and simultaneously overcome the limited growth.
Protein trafficking and secretory pathway	Engineering the endosome-to-Golgi trafficking can significantly reduce intracellular protein retention and thus accelerate the secretion of the protein. Strengthening anterograde trafficking can also increase the protein secretion.

In depth review of these tools is available in [1-3].

Supplementary Table 8. List of plasmids and strains in this study.

Plasmids / Strains	Description / Genotype	Reference
**Plasmids**		
pJ1214	Expression vector containing *TEF1* promoter, 2μ high-copy origin, *URA3* marker, Amp^R, and high-copy-number pUC origin of replication	ATUM
pD1214-FAKS	pJ1214 vector containing a secretion signal of MFα for secreted expression	ATUM
pSEC-Suc2-tSPF	Expression of tSPF with N-terminal Suc2 secretion signal and a C-terminal 6 × His tag, in pJ1214-FAKS	This study
pSEC-Pho5-tSPF	Expression of tSPF with N-terminal Pho5 secretion signal and a C-terminal 6 × His tag, in pJ1214-FAKS	This study
pSEC-MFα-tSPF	Expression of tSPF with a C-terminal 6 × His tag, in pJ1214-FAKS	This study
pSEC-tSPF	Expression of tSPF with a C-terminal 6 × His tag, in pJ1214	This study
pLM494	Harboring β-carotene biosynthesis genes (*crtE*, *crtI*, *crtYB*, *tHMG1*), CEN/ARS single-copy origin and *URA3* marker	Addgene
p415-GPD	Expression vector containing *GPD* promoter, CEN/ARS single-copy origin and *LEU2* marker	[4]
p415-BC	p415 harboring β-carotene biosynthesis genes (*crtE*, *crtI*, *crtYB*, *tHMG1*)	This study
p426-TEF1	Expression vector containing *TEF1* promoter, 2μ high-copy origin and *URA3* marker	[4]
pSEC-Suc2-tSPF-GFP	Expression of tSPF with N-terminal Suc2 secretion signal and C-terminal GFP with a 6 × His tag, in p426-TEF1	This study
pSEC-Pho5-tSPF-GFP	Expression of tSPF with N-terminal Pho5 secretion signal and C-terminal GFP with a 6 × His tag, in p426-TEF1	This study
pSEC-MFα-tSPF-GFP	Expression of tSPF with N-terminal MFα secretion signal and C-terminal GFP with a 6 × His tag, in p426-TEF1	This study
pSEC-tSPF-GFP	Expression of tSPF fused with GFP at C-terminal with a 6 × His tag, in p426-TEF1	This study
**Strains**		
CEN.PK2-1D	*MATα ura3-52 trp1-289 leu2-3,112 his3Δ1 MAL2-8^C SUC2*	Euroscarf
SQ (SQ03-INO2; referred to as SQ)	CEN.PK2-1D Δ*leu2::P_{TEF1}-ERG20* Δ*lpp1::P_{GPD}tHMG1* Δ*opi1* Δ*dpp1::P_{GPD}tHMG1* Δ*ypl062w::P_{GPD}tHMG1*	[5]
Suc2-tSPF/SQ	SQ [pSEC-Suc2-tSPF]	This study
Pho5-tSPF/SQ	SQ [pSEC-Pho5-tSPF]	This study
MFα-tSPF/SQ	SQ [pSEC-MFα-tSPF]	This study

tSPF/SQ	SQ [pSEC-tSPF]	This study
Suc2-tSPF-GFP/SQ	SQ [pSEC-Suc2-tSPF-GFP]	This study
Pho5-tSPF-GFP/SQ	SQ [pSEC-Pho5-tSPF-GFP]	This study
MFα-tSPF-GFP/SQ	SQ [pSEC-MFα-tSPF-GFP]	This study
tSPF-GFP/SQ	SQ [pSEC-tSPF-GFP]	This study
BC	CEN.PK2-1D [p415-BC]	This study
tSPF/BC	CEN.PK2-1D [p415-BC] [pSEC-tSPF]	This study
Suc2-tSPF/BC	CEN.PK2-1D [p415-BC] [pSEC-Suc2-tSPF]	This study

Supplementary Table 9. List of primers in this study.

Primer name	Primer sequence (5'-3')
SUC2 S.S-mut_F	GGCTGGTTTTGCAGCCAAAATATCTGCAATGGAATTCAGCGGTAGAG TTGG
SUC2 S.S-mut_R	AAAAGGAAAAGGAAAGCTTGCAAAAGCATTTTAAACTTAGATTAGATT GCTATGCTTTC
PHO5 S.S-mut_F	TTTAGCCGCTTCTTTGGCCAATGCAATGGAATTCAGCGGTAGAGTTG G
PHO5 S.S-mut_R	ATTGAATAAACAACAGATTTAAACATTTTAAACTTAGATTAGATTGCTAT GCTTTC
S.S-DEL_F	ATGGAATTCAGCGGTAGAG
S.S-DEL_R	TTTAAACTTAGATTAGATTGCTATGC
Null_tSPF_speI_F	CGGACTAGTATGGAATTCAGCGGTAGAG
MFα_tSPF_speI_F	CTAGAACTAGTATGAGATTCCCATCTATTTTCAC
PHO5_tSPF_speI_F	CGGACTAGTATGTTTAAATCTGTTGTTTATTCAATTTTAGC
SUC2_tSPF_speI_F	CGGACTAGTATGCTTTTGCAAGCTTTCC
tSPF_3xGly_XhoI_R	CCGCTCGAGCCCCCCCCCTTAACTTGATCTCTCACGTAGTAC
GFP_F	GGGTGGTGGTCTCGAGATGTCTAAAGGTGAAGAATTATTCACTG
GFP_R	AATTACATGACTCGAGTCAGTGGTGATGATGGTGATGTTTGTACAATT CATCCATACCATGGGTAATACCAGCAGCAGTAACAAATTCTAAC

Supplementary Table 10. Comparison of our metabolite trafficking system with membrane transporter approach for terpene secretion

Approaches	Host strain	Target terpene	Secreted (intracellular)	Secretion efficiency*
Our approach	*S. cerevisiae*	β-carotene	**1.4 mg/L** (7.65 mg/L)	**23-fold**
		squalene	**226 mg/L** (557 mg/L)	**26-fold**
Transporter	*S. cerevisiae*	β-carotene	**7.04 mg/L** (116.3 mg/L)	**4.04-fold**
	E. coli	β-carotene	**0.25 mg/L** (0.38 mg/cell x 10^{-12})	**4.4-fold**
		Lycopene	**0.21 mg/L** (not reported)	**4.3-fold**

* The ratio of the amount of secretion by the engineered cells for each approach to that by the non-engineered cells.

Supplementary Note 1. Within cells, terpenes are captured by tSPF, but out of the cells, they are released from the tSPF and transferred to dodecane.

Consideration of product properties such as binding affinities and partitioning coefficients would be important for unraveling the secretion mechanism of hydrophobic terpenes such as squalene and β-carotene. Previously, the binding interaction between squalene and SPF has been thoroughly investigated, and the K_d of squalene to tSPF has been reported to be approximately 40.10 μM [6], and by our molecular docking with AutoDock Vina in PyRx (Fig. 3E), we suspected that the K_d of β-carotene to SPF would be comparable with that of squalene. As the intracellular concentration of squalene was 476.9 mg/L (1161.1 μM) at 72-h cultivation, tSPF could efficiently capture the squalene within the cells.

On the other hand, when terpene-loaded tSPFs were secreted, we believe that the terpenes should be mostly in the dodecane due to their significantly high octanol/water partition coefficients. The octanol/water partition coefficients ($log P_{oct/wat}$; from www.chemicalize.org) for squalene and β-carotene are 10.67 and 11.12, respectively, and because of this hydrophobicity, squalene and β-carotene are almost insoluble in aqueous environments like the cytoplasm and growth medium, thus accumulating in cellular membranes [7]. However, when the two-phase cultivation system comprising culture medium with an overlay of a biocompatible organic solvent such as decane and dodecane is employed, the hydrophobic products are simply transferred from the cell membranes to the organic solvents [8-10]. Similar with the product transfer from the membrane to the organic solvent in the previous literature, we expect that most secreted terpenes could be unloaded into the dodecane phase, and our HPLC measurements also confirmed that the secreted terpenes were not found in the growth

medium, but in the dodecane (Fig. 2B, Fig. 3C and Supplementary Fig. 3).

Due to this high extraction efficiency, we believe that downstream processing can be simple; with no harvesting and cell disruption, valuable target metabolites could be simply and easily recovered from biocompatible organic solvents, holding great potential for continuous flow production of high-value products in an efficient and cost-effective manner.

Supplementary Note 2. Loading efficiency of target terpenes by tSPF carrier proteins becomes the terpene concentration-dependent within cells according to thermodynamics of terpene-protein interactions.

To determine loading efficiency of terpene into tSPF, it is necessary to consider the binding affinity between the target terpene and the tSPF carrier protein. Based on thermodynamics of protein-ligand binding, the affinity between a protein and a ligand is described by a dissociation constant (K_d). It has been reported that the K_d of squalene to tSPF is approximately 40.10 μM [6], which means that when the intracellular concentration of target squalene is higher than ~40.10 μM, more than half of tSPF is supposed to capture squalene. When we measured the intracellular concentration of squalene after 144-h cultivation using Suc2-tSPF/SQ, the squalene concentration was 557.03 mg/L (1356.20 μM), which is about 34 times higher than the K_d value, indicating that most overexpressed tSPF carrier proteins could be fully occupied with the squalene products inside the cells and subsequently secreted with the captured terpenes into extracellular spaces.

When low levels of other terpenes such as intermediates and bypass products are maintained in the cells, *i.e.*, much lower intracellular concentrations than their K_ds to tSPF, the only target terpene would be successfully captured by the signal peptide-guided tSPF, thereby achieving the selective target terpene secretion. For example, our squalene-producing cells did not secrete 2,3-oxidosqualene, a metabolic intermediate in the squalene biosynthesis pathway. As the K_d of 2,3-oxidosqualene to tSPF is known to be 5.44 μM [6], the binding affinity of 2,3-oxidosqualene to tSPF is supposed to be even 8 times higher than that of squalene. However, when we investigated the intracellular concentration of 2,3-oxidosqualene after 144-h cultivation using Suc2-tSPF/SQ, the 2,3-oxidosqualene concentration was significantly low (0.74 mg/L = 1.73 μM); this intracellular concentration is quite lower than the K_d value, so loading of 2,3-oxidosqualene into tSPF carrier proteins would be highly limited.

To confirm that this intracellular concentration-dependent terpene secretion mechanism holds true for β-carotene and its lycopene precursor, β-carotene-producing cells were also investigated. As shown in Supplementary Table 5, the intracellular concentration of lycopene precursors was not negligibly small, compared to that of β-carotene products. Our molecular docking simulation anticipated high binding affinities of β-carotene and lycopene to tSPF (Fig. 3E and Supplementary Table 6), suggesting that their comparable concentrations allow both terpenes to be captured and exported by signal peptide-guided tSPF carriers. Whereas 2,3-oxidosqualene with a low intracellular concentration was not secreted by squalene-producing cells, lycopene with a relatively high concentration was secreted by the β-carotene-producing cells; from these different results, we can conclude that secretion selectivity relies on which terpenes are significantly accumulated within the cells by engineering biosynthetic pathways.

Even though the SPF serves as a carrier protein to capture many different hydrophobic molecules, the loading efficiency strongly depends on target concentrations within cells. By metabolic engineering, we can determine which metabolites are accumulated inside the cells; the exclusively accumulated terpene would be preferentially loaded into the SPF carriers, so the signal peptide-guided SPF proteins can actualize the secretion of selectively overproduced terpene controlled by metabolic engineering, *i.e.*, selective terpene secretion.

Supplementary Note 3. Comparison of our metabolite trafficking system by metabolite carrier protein sorting with previous membrane transporter systems by transporter overexpression.

As large, hydrophobic molecules, such as terpenes, cannot be effectively excreted from cells, they exclusively accumulate inside. Biologically, this intracellular accumulation can inhibit corresponding synthesis pathways, affecting cellular physiological functions [11,12]. Commercially, extraction of the large, hydrophobic molecules from the cells would be time-consuming and need a high cost [13], impacting on the economic viability of microbial production for such high-value products [14]. In addressing this critical accumulation issue, transmembrane transporters have been discovered and demonstrated to be used for the efflux of hydrophobic compounds. For instance, “Engineering endogenous ABC transporter with improving ATP supply and membrane flexibility enhances the secretion of β-carotene in *Saccharomyces cerevisiae*” by Xiao Bu *et al.* [15] focused on membrane ATP-binding cassette (ABC) transporters capable of exporting lipids across membranes by ATP hydrolysis [16-19], and

when the membrane ABC transporters were overexpressed, the efflux of membrane-associated β-carotene increased by excessive ATP consumption.

In our study, to enable selective terpene secretion, we explored protein sorting by controlling “terpene-loaded carriers.” Our novel approach is superior to the transporter overexpression approach relevant to “membrane-associated products,” as described below.

First, we willingly choose which terpene products are exclusively secreted from cells. Based on thermodynamics of simple terpene-protein interactions, the target terpenes can be captured by carrier proteins and destined to be secreted out of the cells. Our approach excludes secretion of other molecules, except for the target terpenes delivered by the carrier proteins.

Second, target terpene-binding carrier proteins are simply guided by export signal peptides, so the large, hydrophobic molecules can readily pass through otherwise impermeable membranes. As the transporter overexpression approach relies on noncognate transport proteins or channels, the control of the transporters by excessive ATP consumption has been known to be significantly challenging. In particular, the overexpression of transporters embedded in the cell membranes would increase the rigidity of the cell membranes and disturb their normal functions, thereby decreasing the cell growth and the transport capacity [20,21]. In contrast, our flexible approach is the cognate secretion pathway *via* co-translational translocation, a nature’s well-established sorting mechanism, so without membrane reconstruction and function disturbance, target terpenes loaded in carrier proteins are efficiently secreted out of host cells.

Third, the biological synthesis of target terpenes and carrier proteins and their

complex formation are spatiotemporally identical, which is highly advantageous for significant improvement of terpene secretion. The transporter overexpression approach demands target molecules, which is synthesized in ER, to pass through hydrophilic cytosols to reach outer membranes, and the voyage of the large, hydrophobic molecules in aqueous environments would not be preferable. In contrast, our approach induces target terpenes (*e.g.*, squalene and β-carotene) and carrier proteins to be synthesized in the ER at the same time and to be bound each other at the same location. As a result, we reported the highest level of terpene secretion to date; for example, while the ABC transporter overexpression strategy showed a 4-fold increase of β-carotene in *S. cerevisiae* compared to control cells, our terpene secretion strategy achieved a 26-fold increase of squalene and a 23-fold increase of β-carotene in *S. cerevisiae* (see Supplementary Table 10).

Fourth, we emphasize the wide applicability of our approach wherein we systematically coupled a target-binding protein with a sorting signal peptide. When cytoplasmic proteins are encrypted with sorting signals, the address labels determine the locations (*e.g.*, intracellular compartments and extracellular milieu) that the proteins can be accurately delivered to. Many kinds of metabolite-binding proteins can be readily fused with desired signal peptides, thereby tailoring different metabolite trafficking pathways in microorganisms.

As this work is a proof-of-concept study, the level of target terpene's extracellular secretion should be further improved in prevailing over that of its intracellular accumulation. However, we realized an unprecedented metabolite secretion pathway by target terpene-loaded carrier protein sorting, and importantly, squalene secretion was reported for the first time in this study to the best of our knowledge. Thus, we

strongly believe that our carrier protein sorting-based strategy for metabolite trafficking is novel and significant to impact on the economic viability and compatibility of highly valuable, yet membrane-impermeable products.

Supplementary Note 4. Possibility for the formation of squalene-SPF complexes with an uneven stoichiometry.

When 1:1 Langmuirian binding model between a SPF and a terpene and 100% binding efficiency is assumed, the terpene-loaded SPF should contain equal amounts of both terpene and SPF; secretion of 1.7 mM squalene (~700 mg/L of squalene) requires production of 1.7 mM SPF (53.7 g/L) at least. When the protein production prowess of yeast cells is considered, such significantly large amounts of SPF proteins would be unlikely to be produced by our engineered yeast cells. Moreover, when we measured the amount of overexpressed tSPF carriers for the squalene-producing yeast cells, it was <1 g/L (~0.032 mM) for 72-h of cultivation, and this amount of tSPF is approximately more than 10 times less than that of squalene (166.62 mg/L = ~0.406 mM) at this cultivation time. However, in understanding the mechanism of carrier protein-assisted secretion, we question if the fundamental assumption of 1:1 stoichiometry between a SPF and a terpene is valid.

In addressing this question, it should be focused that that the binding between the SPF and the terpene appears to be dominantly driven by hydrophobic interactions. It is well known that nonpolar compounds tend to cluster together in an aqueous solution by excluding water molecules [22,23]. As the octanol/water partitioning coefficient ($logP_{oct/wat}$) and solubility of squalene is 10.67 and 0.124 mg/L, respectively [24], a

squalene molecule cannot exist alone in water, but squalene clusters should be formed instead, which would be thermodynamically favorable [24,25]. This means that when a ligand binding cavity within the SPF is accessed by squalene, the structure of squalene at a molecular level is likely to be a cluster, rather than a single molecule. Even if a single molecule is captured by SPF, the complex of SPF and squalene is eager to recruit more squalene molecules to minimize the surface area to volume ratio due to the hydrophobic effect [25,26]. Thus, the assumption of 1:1 stoichiometry between a SPF and a terpene may be invalid, and a SPF carrier protein may carry multiple squalene molecules.

Using electrospray ionization-mass spectrometry (ESI-MS), we observed the possibility for the formation of squalene-SPF complexes with an uneven stoichiometry. Considering the reported K_d of squalene to tSPF (~40.1 μM), we incubated 2 μM tSPF with 100 μM squalene to ensure 100% complex formation, and complex stoichiometries were investigated by analyzing the obtained ESI-mass spectrum (Supplementary Fig. 10). The mass-to-charge ratio (m/z) distribution of tSPF-squalene complex revealed that one tSPF is in complex with 4-5 squalene molecules, as we calculated the number of squalene molecules bound to a tSPF protein with their mass. Since the detachment of several squalene molecules can occur during the ESI process and gas-phase transmission, the number of bound squalene molecules in solution phase may be larger than five. Similar with our observation, there have been several studies that reported multimeric assembly of lipid-binding proteins with hydrophobic molecules [27,28].

Additionally, we performed confocal fluorescence microscopy experiments to find the evidence of terpene-tSPF complexes with an uneven stoichiometry (Supplementary

Fig. 11). During squalene secretion by the engineered yeast strain (Suc2-tSPF-expressing SQ, Suc2-tSPF/SQ), the green and red fluorescence by GFP-fused tSPF and Nile red-stained squalene overlapped each other (Supplementary Fig. 11A), indicating the location of tSPF and squalene would be the same before secretion, presumably by formation of tSPF-squalene droplet clusters. Even in the extracellular milieu, the fluorescent overlapping was also observed, suggesting that even after secretion, tSPF and squalene would stay together, forming tSPF-squalene droplets. In terms of squalene-tSPF complexes with an uneven stoichiometry, we intentionally prepared squalene droplets (100 ~ 400 nm in diameter) and mixed with GFP-fused tSPFs of which concentration (~0.1 mg/mL) was 100 times less than that of squalene (~10 mg/mL) (Supplementary Fig. 11B). As a result, the locations of squalene droplets and tSPFs were observed to be the same, indicating that the tSPF protein would bind not only to the single molecule of squalene but also to the squalene cluster. We note that without tSPF fusion, the GFP proteins did not bind to the squalene droplets.

Although there still remains a gap in fully understanding the mechanism of SPF-driven terpene secretion, the observations by ESI-MS and confocal fluorescence microscopy are considered to support our claim, "tSPF is supposed to form squalene clusters and carry multiple squalene molecules due to a driving force of hydrophobic interactions." We note again that the tSPF overexpressed by yeast cells experimentally secreted squalene of which amount is >10 times larger than that of tSPF. However, the effect of crowding in biological environments [26,29] is known to promote formation of biomolecular condensates, and this infers that somehow the tSPF binding ratio of squalene in the ER may be able to be higher than that of *in vitro* experiment.

Supplementary Note 5. Incomplete vs complete secretion of intracellular terpenes.

The reason why intracellular terpenes cannot be fully secreted in our study is because the amount of overexpressed tSPF carrier proteins is not sufficient to load all the amount of produced terpenes within cells. The binding interaction between tSPF and squalene is quite strong; when we consider the K_d of squalene to tSPF (~40.1 μM) and the intracellular squalene concentration (~1161.1 μM = 476.9 mg/L at 72-h cultivation), the tSPF carrier proteins are always fully loaded with squalene before subsequent secretion events. Thus, the expression level of tSPF should be the main limiting factor that constrains the secretion level of squalene.

The potential solution to increase the secretion level of terpenes can be to further increase the expression level of tSPF carrier proteins. Currently, to make the squalene-producing cells secrete 166.62 mg/L squalene (~406 μM), the yeast cells required to express ~1 g/L tSPF carriers. *Pichia pastoris* has been demonstrated to produce even 35 g/L of albumin [30,31], which highly encourages us to further improve our terpene secretion approach by maximizing the yield of tSPF production. Furthermore, one recent study suggested that engineering the protein secretory pathway of *S. cerevisia*e enables improved protein production and secretion to gram per liter level [32]; modifications of the endosome-to-Golgi trafficking and anterograde trafficking were found to effectively reduce the intracellular retention of the desired protein and thus increase its secretion. We envision that if the tSPF expression and secretion level is several times higher than the current one, it may be possible to secrete the terpene products from the cells more efficiently.

Even though the terpene products are partially exported from the engineered cells,

the incomplete secretion can be still useful. For example, even if desired products or by-products are toxic to inhibit host cells' growth and biological functions, they can be often tolerated to a certain level. In maintaining homeostasis or regulating overflow metabolism, the host cells could not require to secrete all the toxic products or by-products; only with their incomplete secretion, the engineered cells would function well or even better. As an exemplar, we observed that even though ~29% of produced squalene was secreted only, the total squalene production increased by ~60%.

When we investigated the result of semi-continuous fermentation (Fig. 2F), the secreted squalene titers increased steadily with time. Assuming that the engineered cells capable of secreting products are well-nourished without aging and mutation, cell cultivation and secreted product collection should be in a continuous manner without cell harvesting or disruption, which would be technically simpler, easier, and more cost-effective than conventional batch fermentation and extraction methods.

Encouraged by the potential of this proof-of-concept work, we aim to develop a more efficient system enabling continuous flow production of valuable metabolites; to do so, our following works would focus on achieving complete secretion of desired metabolites, and one of its possible solutions could be maximizing the expression and secretion of carrier proteins.

Supplementary Note 6. Possible influences induced by tSPF overexpression and transport in the cells.

Overexpression of target proteins causes cell resources to be hijacked, making cells damaged in several ways, for example, by exhausting cell resources to make other

proteins (known as *the protein burden*) or by overloading transport systems that take various proteins to specific cell compartments (*transport overload*), or by ultimately upsetting balanced cellular processes [33-35]. In this study, overexpression of the tSPF fused with or without signal peptides appeared to repress the cell growth when compared to no overexpression of tSPF. We reason that the tSPF overexpression was initiated from the strongest yeast *TDH3* promoter on the high-copy number plasmid, thereby causing growth defects. It is broadly interpreted that the forced overexpression of a protein often leads to the reduced cell growth (OD600), which is also well-known as the *protein burden* effect or the cost of protein production.

With regard to the signal peptide-fused, tSPF-expressing cells, the OD600 of the cells with large terpene secretion (Suc2-tSPF/SQ and Pho5-tSPF/SQ) showed no distinct difference with that of the cells with small terpene secretion (MFα-tSPF/SQ) after 72-h cultivation. However, the notable decrease in OD600 was observed from the cells mediated by Suc2-tSPF or Pho5-tSPF, compared to the cells by MFα-tSPF after 144-h cultivation. In detail, among the three different signal peptides (Suc2, Pho5, and MFα), Suc2 transported tSPF most largely, which was consistent with the finding that Suc2-tSPF hindered the cell growth most significantly; this result indicates that *transport overload* of overexpressed tSPF carrier proteins can hinder cell growth, resulting in low OD600. The cell growth (OD600) could therefore be influenced though the diverse cost induced by tSPF overexpression and transport in the cells.

Supplementary References

1 Dragosits, M., Nicklas, D. & Tagkopoulos, I. A synthetic biology approach to self-regulatory recombinant protein production in *Escherichia coli*. *J Biol Eng* **6**, 2, doi:10.1186/1754-1611-6-2 (2012).

2 Jana, S. & Deb, J. K. Strategies for efficient production of heterologous proteins in *Escherichia coli*. *Appl Microbiol Biotechnol* **67**, 289-298, doi:10.1007/s00253-004-1814-0 (2005).

3 Yang, D., Park, S. Y., Park, Y. S., Eun, H. & Lee, S. Y. Metabolic Engineering of *Escherichia coli* for Natural Product Biosynthesis. *Trends Biotechnol* **38**, 745-765, doi:10.1016/j.tibtech.2019.11.007 (2020).

4 Lee, J. Y., Kang, C. D., Lee, S. H., Park, Y. K. & Cho, K. M. Engineering cellular redox balance in *Saccharomyces cerevisiae* for improved production of L-lactic acid. *Biotechnol Bioeng* **112**, 751-758, doi:10.1002/bit.25488 (2015).

5 Kim, J. E. *et al.* Tailoring the *Saccharomyces cerevisiae* endoplasmic reticulum for functional assembly of terpene synthesis pathway. *Metab Eng* **56**, 50-59, doi:10.1016/j.ymben.2019.08.013 (2019).

6 Christen, M. *et al.* Structural insights on cholesterol endosynthesis: Binding of squalene and 2,3-oxidosqualene to supernatant protein factor. *J Struct Biol* **190**, 261-270, doi:10.1016/j.jsb.2015.05.001 (2015).

7 Meckel, R. & Kester, A. Extractability of carotenoid pigments from non-photosynthetic bacteria with solvents and detergents: Implications for the location and binding of the pigments. *Microbiology* **120**, 111-116 (1980).

8 Dunlop, M. J. *et al.* Engineering microbial biofuel tolerance and export using efflux pumps. *Molecular systems biology* **7**, 487 (2011).

9 Peralta-Yahya, P. P. *et al.* Identification and microbial production of a terpene-based advanced biofuel. *Nat Commun* **2**, 483, doi:10.1038/ncomms1494 (2011).

10 Yoon, K. W., Doo, E. H., Kim, S. W. & Park, J. B. In situ recovery of lycopene during biosynthesis with recombinant *Escherichia coli*. *J Biotechnol* **135**, 291-294, doi:10.1016/j.jbiotec.2008.04.001 (2008).

11 Ahrazem, O. *et al.* The carotenoid cleavage dioxygenase CCD 2 catalysing the synthesis of crocetin in spring crocuses and saffron is a plastidial enzyme. *New Phytologist* **209**, 650-663 (2016).

12 Wu, T. *et al.* Membrane engineering - A novel strategy to enhance the production and accumulation of beta-carotene in *Escherichia coli*. *Metab Eng* **43**, 85-91, doi:10.1016/j.ymben.2017.07.001 (2017).

13 Cadoni, E., De Giorgi, M. R., Medda, E. & Poma, G. Supercritical CO2 extraction of lycopene and β-carotene from ripe tomatoes. *Dyes and pigments* **44**, 27-32 (1999).

14 Wu, G. *et al.* Metabolic Burden: Cornerstones in Synthetic Biology and Metabolic Engineering Applications. *Trends Biotechnol* **34**, 652-664, doi:10.1016/j.tibtech.2016.02.010 (2016).

15 Bu, X. *et al.* Engineering endogenous ABC transporter with improving ATP supply and membrane flexibility enhances the secretion of beta-carotene in *Saccharomyces cerevisiae*. *Biotechnol Biofuels* **13**, 168, doi:10.1186/s13068-020-01809-6 (2020).

16 Chen, B., Ling, H. & Chang, M. W. Transporter engineering for improved tolerance against alkane biofuels in *Saccharomyces cerevisiae*. *Biotechnol Biofuels* **6**, 21, doi:10.1186/1754-6834-6-21 (2013).

17 Claus, S., Jezierska, S. & Van Bogaert, I. N. A. Protein-facilitated transport of hydrophobic molecules across the yeast plasma membrane. *FEBS Lett* **593**, 1508-1527, doi:10.1002/1873-3468.13469 (2019).

18 Doshi, R., Nguyen, T. & Chang, G. Transporter-mediated biofuel secretion. *Proc Natl Acad Sci U S A* **110**, 7642-7647, doi:10.1073/pnas.1301358110 (2013).

19 Lee, J. J., Chen, L., Cao, B. & Chen, W. N. Engineering Rhodosporidium toruloides with a membrane transporter facilitates production and separation of carotenoids and lipids in a bi-phasic culture. *Appl Microbiol Biotechnol* **100**, 869-877, doi:10.1007/s00253-015-7102-3 (2016).

20 Jamin, N., Garrigos, M., Jaxel, C., Frelet-Barrand, A. & Orlowski, S. Ectopic Neo-Formed Intracellular Membranes in *Escherichia coli*: A Response to Membrane Protein-Induced Stress Involving Membrane Curvature and Domains. *Biomolecules* **8**, doi:10.3390/biom8030088 (2018).

21 Krasowska, A., Lukaszewicz, M., Bartosiewicz, D. & Sigler, K. Cell ATP level of *Saccharomyces cerevisiae* sensitively responds to culture growth and drug-inflicted variations in membrane integrity and PDR pump activity. *Biochem Biophys Res Commun* **395**, 51-55, doi:10.1016/j.bbrc.2010.03.133 (2010).

22 Brini, E. *et al.* How water's properties are encoded in its molecular structure and energies. *Chemical reviews* **117**, 12385-12414 (2017).

23 Kumar, A. Salt effects on Diels-Alder reaction kinetics. *Chem Rev* **101**, 1-19, doi:10.1021/cr990410+ (2001).

24 Tetko, I. V. *et al.* Virtual computational chemistry laboratory--design and description. *J Comput Aided Mol Des* **19**, 453-463, doi:10.1007/s10822-005-8694-y (2005).

25 Tadros, T., Izquierdo, P., Esquena, J. & Solans, C. Formation and stability of nano-emulsions. *Adv Colloid Interface Sci* **108-109**, 303-318, doi:10.1016/j.cis.2003.10.023 (2004).

26 Ralston, G. Effects of" crowding" in protein solutions. *Journal of chemical education* **67**, 857 (1990).

27 Laganowsky, A. *et al.* Membrane proteins bind lipids selectively to modulate their structure and function. *Nature* **510**, 172-175 (2014).

28 Liu, Y. *et al.* Selective binding of a toxin and phosphatidylinositides to a mammalian potassium channel. *Nat Commun* **10**, 1352, doi:10.1038/s41467-019-09333-4 (2019).

29 van den Berg, J., Boersma, A. J. & Poolman, B. Microorganisms maintain crowding homeostasis. *Nat Rev Microbiol* **15**, 309-318, doi:10.1038/nrmicro.2017.17 (2017).

30 Celik, E. & Calik, P. Production of recombinant proteins by yeast cells. *Biotechnol Adv* **30**, 1108-1118, doi:10.1016/j.biotechadv.2011.09.011 (2012).

31 Kazemali, M. R. *et al.* Enhanced truncated-t-PA (CT-b) expression in high-cell-density fed-batch cultures of *Pichia pastoris* through optimization of a mixed feeding strategy by response surface methodology. *Bioprocess Biosyst Eng* **39**, 565-573, doi:10.1007/s00449-016-1538-4 (2016).

32 Huang, M., Wang, G., Qin, J., Petranovic, D. & Nielsen, J. Engineering the protein secretory pathway of *Saccharomyces cerevisiae* enables improved protein production. *Proc Natl Acad Sci U S A* **115**, E11025-E11032, doi:10.1073/pnas.1809921115 (2018).

33 Bolognesi, B. & Lehner, B. Protein overexpression: reaching the limit. *Elife* **7**, e39804 (2018).

34 Eguchi, Y. *et al.* Estimating the protein burden limit of yeast cells by measuring

the expression limits of glycolytic proteins. *Elife* **7**, doi:10.7554/eLife.34595 (2018).

35 Kafri, M., Metzl-Raz, E., Jona, G. & Barkai, N. The Cost of Protein Production. *Cell Rep* **14**, 22-31, doi:10.1016/j.celrep.2015.12.015 (2016).
