# Supplementary material for: Metabolite trafficking enables membrane-impermeable-terpene secretion by yeast
Source: Nat Commun. 2022 May 11;13:2605. doi: 10.1038/s41467-022-30312-9 (PMC9095633; doi:10.1038/s41467-022-30312-9)
Supplement: Supplementary file 3 — Reporting Summary [file 41467_2022_30312_MOESM3_ESM.pdf]

nature research

Corresponding author(s): Ju Young Lee, Seung Soo Oh

Last updated by author(s): Mar 8, 2022

Reporting Summary

Nature Research wishes to improve the reproducibility of the work that we publish. This form provides structure for consistency and transparency in reporting. For further information on Nature Research policies, see our Editorial Policies and the Editorial Policy Checklist.

Statistics

For all statistical analyses, confirm that the following items are present in the figure legend, table legend, main text, or Methods section.

n/a	Confirmed	
☐	☒	The exact sample size (*n*) for each experimental group/condition, given as a discrete number and unit of measurement
☐	☒	A statement on whether measurements were taken from distinct samples or whether the same sample was measured repeatedly
☒	☐	The statistical test(s) used AND whether they are one- or two-sided<br>*Only common tests should be described solely by name; describe more complex techniques in the Methods section.*
☒	☐	A description of all covariates tested
☒	☐	A description of any assumptions or corrections, such as tests of normality and adjustment for multiple comparisons
☐	☒	A full description of the statistical parameters including central tendency (e.g. means) or other basic estimates (e.g. regression coefficient) AND variation (e.g. standard deviation) or associated estimates of uncertainty (e.g. confidence intervals)
☒	☐	For null hypothesis testing, the test statistic (e.g. *F*, *t*, *r*) with confidence intervals, effect sizes, degrees of freedom and *P* value noted<br>*Give P values as exact values whenever suitable.*
☒	☐	For Bayesian analysis, information on the choice of priors and Markov chain Monte Carlo settings
☒	☐	For hierarchical and complex designs, identification of the appropriate level for tests and full reporting of outcomes
☒	☐	Estimates of effect sizes (e.g. Cohen's *d*, Pearson's *r*), indicating how they were calculated

Our web collection on statistics for biologists contains articles on many of the points above.

Software and code

Policy information about availability of computer code

Data collection	Agilent OpenLAB CDS ChemStation C.01.07 SR1, ChemiDoc MP imaging system
Data analysis	ZEN imaging software 2.1, Image-Pro PLUS 7.0.1, Discovery Studio 2020 v20.1.0.19, Computer Atlas of Surface Topology of protein 3.0 (CASTp), AutoDock Vina 1.1.2 in PyRx 0.8

For manuscripts utilizing custom algorithms or software that are central to the research but not yet described in published literature, software must be made available to editors and reviewers. We strongly encourage code deposition in a community repository (e.g. GitHub). See the Nature Research guidelines for submitting code & software for further information.

Data

Policy information about availability of data

All manuscripts must include a data availability statement. This statement should provide the following information, where applicable:

- Accession codes, unique identifiers, or web links for publicly available datasets
- A list of figures that have associated raw data
- A description of any restrictions on data availability

All data in main text and supplementary information are available from the corresponding authors upon reasonable request. The data for molecular docking are from the Protein Data Bank (PDB accession code: 4OMK) and the PubChem compound database under accession codes: 5366020, 5319723, 5320250, 6549, 637566, 643820, 22311, 1254, 6654, 2537, 10364, 7439, 5281517, 5284507, 10104370, 9855795, 11052747, 161937, 9548702, 68827, 12523, 5280435, 101750, 445354, 9548711, 182677, 9548698, 6857485, 154992, 9548699, 9548828, 638072, 246983, 73170, 73145, 10115, 10494, 446925, 5281252, 5280489, 5281243, 5280899, 5281224, 33032 and 107735. The processed data used to generate figures are provided in the Source Data file. Source data are provided with this paper.

Field-specific reporting

Please select the one below that is the best fit for your research. If you are not sure, read the appropriate sections before making your selection.

☒ Life sciences ☐ Behavioural & social sciences ☐ Ecological, evolutionary & environmental sciences

For a reference copy of the document with all sections, see nature.com/documents/nr-reporting-summary-flat.pdf

Life sciences study design

All studies must disclose on these points even when the disclosure is negative.

Sample size	Sample size of at least three was chosen following all previous publications in similar field. The sample size is visible in the graphs by individual data points and is stated in each figure legend. Published examples are Braus, S.A.G. et al., Nat Commun. (2022) https://doi.org/10.1038/s41467-022-28690-1 or Makino et al., Nat Commun. (2022) https://doi.org/10.1038/s41467-021-22574-6.
Data exclusions	No data were excluded from analysis.
Replication	All experiments were independently reproduced at least three times. Replicate experiments were successful.
Randomization	Randomization was not applicable in this study because there are no static test that requires randomization of samples.
Blinding	Blinding was not performed for this study because this is not required in this field. All data were analyzed and checked by multiple authors and reviewed by the corresponding authors.

Reporting for specific materials, systems and methods

We require information from authors about some types of materials, experimental systems and methods used in many studies. Here, indicate whether each material, system or method listed is relevant to your study. If you are not sure if a list item applies to your research, read the appropriate section before selecting a response.

Materials & experimental systems

n/a	Involved in the study	
☐	☒	Antibodies
☒	☐	Eukaryotic cell lines
☒	☐	Palaeontology and archaeology
☒	☐	Animals and other organisms
☒	☐	Human research participants
☒	☐	Clinical data
☒	☐	Dual use research of concern

Methods

n/a	Involved in the study	
☒	☐	ChIP-seq
☒	☐	Flow cytometry
☒	☐	MRI-based neuroimaging

Antibodies

Antibodies used	anti-actin antibody (Chemicon International Inc., MA, USA, Catalog number: MAB1501, clone C4, RRID:AB_2223041), anti-His antibody (Santa Cruz Biotechnology, Inc., CA, USA, Catalog number: sc-8036, H-3, RRID:AB_627727), peroxidase-conjugated Goat anti-Mouse IgG antibody (Jackson Immun. Lab., USA, Catalog number: 115-035-003, RRID:AB_10015289)
Validation	Anti-actin antibody (ELISA and western blot), anti-His antibody (western blot), and peroxidase-conjugated Goat anti-Mouse IgG antibody (immunoelectrophoresis and ELISA) were either published and established or validated by the manufacturer.

April 2020
